# The Association between Total Protein, Animal Protein, and Animal Protein Sources with Risk of Inflammatory Bowel Diseases: A Systematic Review and Meta-Analysis of Cohort Studies

**DOI:** 10.1016/j.advnut.2023.05.008

**Published:** 2023-05-14

**Authors:** Sepide Talebi, Sheida Zeraattalab-Motlagh, Mehran Rahimlou, Fatemeh Naeini, Mahsa Ranjbar, Ali Talebi, Hamed Mohammadi

**Affiliations:** 1 Students’ Scientific Research Center (SSRC), Tehran University of Medical Sciences, Tehran, Iran; 2 Department of Clinical Nutrition, School of Nutritional Sciences and Dietetics, Tehran University of Medical Sciences, Tehran, Iran; 3 Department of Community Nutrition, School of Nutritional Sciences and Dietetics, Tehran University of Medical Sciences, Tehran, Iran; 4 Department of Nutrition, Faculty of Medicine, Zanjan University of Medical Sciences, Zanjan, Iran; 5 Clinical Pharmacy Department, Faculty of Pharmacy, Tehran University of Medical Sciences, Tehran, Iran

**Keywords:** animal protein, dairy, fish, inflammatory bowel diseases, meat, meta-analysis, nutrition, poultry, systematic review

## Abstract

We aimed to conduct this dose-dependent meta-analysis to examine the relation between total protein, animal protein, and its sources with inflammatory bowel disease (IBD). We searched databases, comprising PubMed/Medline, Web of Science (ISI), Embase, and Google Scholar, for the published studies up to 28 March 2023. Prospective cohort study designs that investigated associations between dietary intake of various animal protein sources and with risk of IBD in the general population were identified. Eleven prospective cohort studies with 4,302,554 participants and 8067 cases were considered eligible. Findings indicated that higher intake of dairy was significantly associated with a lower risk of IBD (relative risk [RR]: 0.81; 95% confidence interval [CI]: 0.72, 0.90), Crohn disease (RR: 0.69; 95% CI: 0.56, 0.86), and ulcerative colitis (RR: 0.84; 95% CI: 0.75, 0.94). There was no association between different sources of animal protein and the risk of IBD. The dose-response analysis suggested that each 100 g/d increment in dietary total meat consumption was associated with a 38% greater risk of IBD. Moreover, a positive linear association was found between total meat intake and risk of IBD (*P*_nonlinearity_ = 0.522, *P*_dose-response_ = 0.005). Overall, among the dietary sources of protein, the risk of IBD increased only with increasing total meat intake, and the consumption of protein from dairy products was found to be a protective factor against the IBD risk.

This trial was registered at PROSPERO as CRD42023397719.

**Statement of Significance**

To our knowledge, this is the first dose-response meta-analysis of prospective cohort studies to assess the relationship between the amount of consumed dietary total protein, animal protein, and animal protein sources and the risk of developing inflammatory bowel disease in the adult population.

## Introduction

Inflammatory bowel disease (IBD) is specified by recurrent inflammation of the intestinal tract that needs life-long supervision [1]. Crohn disease (CD) and ulcerative colitis (UC) are 2 predominant forms of IBD [2]. Although CD can lead to destruction anywhere along the digestive tract (across the mouth and anus), UC is mainly limited to the colon and rectum [3,4]. During the past several decades, the prevalence and incidence of IBD have exceeded globally. It is considered that ∼1.5 and 3 million people in United States and Europe, as well as several thousand people in other regions over the globe, are affected [5,6]. Comparable with other intestinal tract disorders, IBD imposes a vast economic burden on society and adversely affects the quality of life [7].

Dysregulated immune response, genetic susceptibility, gut microbiome, and environmental factors may play a part in IBD development, but they failed to indicate sufficient information regarding dietary triggers of IBD [8,9]. Generally, the role of the total protein, as well as animal protein and its sources, in IBD has been studied to understand which ones need to be avoided [10,11].

Narula et al. [12], in a review of cohort studies, revealed that higher ultraprocessed food (e.g., processed meat) and lower unprocessed food consumption (e.g., milk, chicken, and egg) were related to an increased risk of CD but not including UC. However, a meta-analysis that analyzed data from observational studies in children and adults showed that higher protein dietary intake was not related to the risk of IBD [11]. In addition, a review study showed no significant relation between higher dietary meat intake and CD or UC risk [13]. The findings from a large European cohort study indicated no relation between the consumption of both total or certain types of dairy products (cheese, milk, or yogurt) and the risk of CD or UC. Nevertheless, subjects who consumed milk had a lower incidence of progressing CD than nonconsumers [14]. Moreover, dietary total and animal protein, as well as animal protein sources intake, has also been evaluated widely in association with developing the chance of chronic diseases [15,16]; however, no decisive evidence is obtained regarding its role in patients with IBD.

These controversial results might be due to the fact that previous relevant reviews have mostly concentrated on results from case-control studies [10,11,17] or conducted on a wide range of age groups (including both children and adults) [11]. In addition, the dose-response meta-analysis has not been studied yet; as a result, their findings could be inconclusive. Thus, we aimed to conduct this dose-dependent meta-analysis to examine the relation between total protein, as well as animal protein and its sources with IBD.

## Methods

We pursued the frameworks specified in the Cochrane Handbook for Systematic Reviews to implement this meta-analysis [18]. Besides, the PRISMA was implemented to declare this review [19]. The protocol of this article was registered on PROSPERO (CRD42023397719).

### Search strategy

We searched online databases, comprising Web of Science (ISI), PubMed/Medline, Embase, and Google Scholar, for the published studies up to 28 March 2023. A literature search was performed and expanded (S.T.), and 2 investigators (M.R. and F.N.) screened the titles/abstracts. The same 2 investigators separately evaluated the relevant full texts for eligibility. Discrepancies were addressed by consensus. Moreover, the references of published observational reviews on the relation between total protein, as well as animal protein, and its sources with the incidence of IBD were manually screened. We did not enforce any publication time or language restrictions. Our comprehensive search strategy is recorded in Supplemental Table 1.

### Eligibility criteria

Two of the investigators (S.T. and M.R.) reviewed the title/abstracts of each article discovered in the literature search to determine articles that: *1*) included general adult study participants (aged ≥18 y); *2*) utilized prospective cohort design; *3*) reported exposure as the consumption of total protein, as well as animal protein, and its sources (e.g., total dairy, milk, meat, fish, poultry, process meat, and egg) in >2 categories; *4*) reported the outcome as the incidence rate of IBD (CD or UC); and *5*) reported suitable effect estimates, including RR, HR, or OR and their 95% CIs.

If more than one cohort study indicated similar data, we selected the ones with thorough records for dose-dependent analysis (for instance, those that reported exposures as categories and indicated adequate details within categories). Other than that, the articles with the largest sample sizes were chosen. We excluded articles with cross-sectional or case-control study designs, as well as publications conducted among patients with all gastrointestinal disorders other than IBD (CD or UC), such as celiac disease, gastroesophageal reflux disease, irritable bowel syndrome, small intestinal bacterial overgrowth, gallstones, and so on.

### Data extraction and quality assessment

The relevant data extracted from all studies by 2 independent investigators (S.T. and M.R.) were outlined as follows: the first author, cohort name, location of study, year of publication, confounders adjusted, follow-up length, instruments used to assess total protein, as well as animal protein, and its sources, number of participants/cases, mean age, method of recognizing outcome, type of exposure, comparison categories and relevant effect sizes (RR, HR, and OR) with their 95% CIs. Any discrepancies were addressed by a discussion with the third investigator (H.M.).

We performed a quality assessment of cohort studies applying the Risk Of Bias In Non-randomized Studies-of Interventions tool [20]. Evaluation of the quality and possible biases related to included studies has been performed by this tool developed by Cochrane [21]. Two investigators (S.T. and F.N.) separately assessed the quality of cohort studies. Discrepancies in the quality assessment were addressed through discussion (Supplemental Table 2).

### Statistical methods

We chose the RRs with 95% CI as the effect estimate for our meta-analysis. The reported HRs were deemed equal to RRs [22]. For cohort studies that demonstrated effect estimates as ORs, we deemed them equivalent to RRs when ORs ranged between 0.5 and 2.5 or when the IBD incidence was low (<10%); if not, we changed them to RR based on the approach of Zhang and Yu [23]. We applied the random effect model to generate the pooled RRs and 95% CIs of IBD risk related to the highest compared with lowest categories of dietary intakes of total protein, as well as animal protein, and its sources. Between-study heterogeneity was assessed by conducting the Cochran *Q* test and *I*^2^ statistic [24].

We did a subgroup analysis according to the region, sex, length of study duration, number of overall participants, and subjects with IBD, dietary assessment, case ascertainment (method of recognizing patients with IBD), and adjusting for confounders, comprising physical activity, sex, BMI, smoking status, EI, and consumption of alcohol. We also conducted meta-regression analyses to discover the potential source of heterogeneity. Publication bias was estimated by Egger’s test and Begg’s test [25]. We carried out a sensitivity analysis after excluding each study at a time to estimate the relative effect of any survey on the summarized effect size.

For linear dose-dependent analysis, the method developed by Greenland and Longnecker [26] and the generally available Stata command documented by Orsini et al. [27] were used. Summary RRs with 95% CIs were estimated for 100 g/d increments in intake of total protein and animal protein, 100 g/d increments in dietary whole and red meat intake, as well as poultry intake, 15 g/d (105 g/wk, approximately equal to a 1 serving/wk) increments in dietary fish, 200 g/d increments in dairy consumption, and 1 egg per d (approximately equal to 50 g/d) increases in egg intake. We subsequently indicated a random effect model to combine each study’s findings.

To conduct our analysis, we deemed the median of each protein category, the numbers of subject or a person year, the number of subjects with IBD, and adjusted effect sizes within a minimum of 2 classes of exposures were pooled from every study. Moreover, most studies ranked protein intake as tertiles, quartiles, or quintiles; as a result, the highest protein intake category (tertiles, quartiles, or quintiles) compared with the lowest category was compiled for analysis. For cohort studies that documented effect estimates per specific increment in the quantity of exposure, the log effect estimate was exponentiated by multiplying the cohort study-certain consumption of the exposure to get the effect estimate for 1 extra serving of exposure [28,29]. When cohort studies described exposures as ranges, the estimated median values were computed by applying the mean of the lower and upper limits. We considered the width of the open categories equivalent to the adjacent category. In every study, we combined effect sizes for both sexes using the fixed effect model when studies demonstrated RRs for women and men separately. On the basis of the manner indicated by Hamling et al. [30], we considered the lowest category in the act of reference group for cohort studies that did not deem the lowest class in consideration of reference group.

In addition, we estimated curve linear or nonlinear dose-dependent relation between total protein, as well as animal protein, and its sources and IBD incidence by applying the restricted cubic line that was made up of 3 nodes at the fixed percentiles (10th, 50th, and 90th) [31]. The correlation across every category of provided RRs was computed, and a 1-stage linear mixed effect model was applied to pool the estimates associated with every cohort study [32]. All analyses were performed by Stata software, version 16.0. *P* value of <0.05 was regarded to be significant.

### Certainty of the evidence

Rating the evidence was performed by applying the updated Grading of Recommendations, Assessment, Development, and Evaluations (GRADE) framework [33,34]. GRADE ranks the evidence as “high,” “moderate,” “low,” and “very low.” The reviewers (S.T. and S.R.M.) separately conducted GRADE judgments.

## Results

### Literature search

**FIGURE 1:**
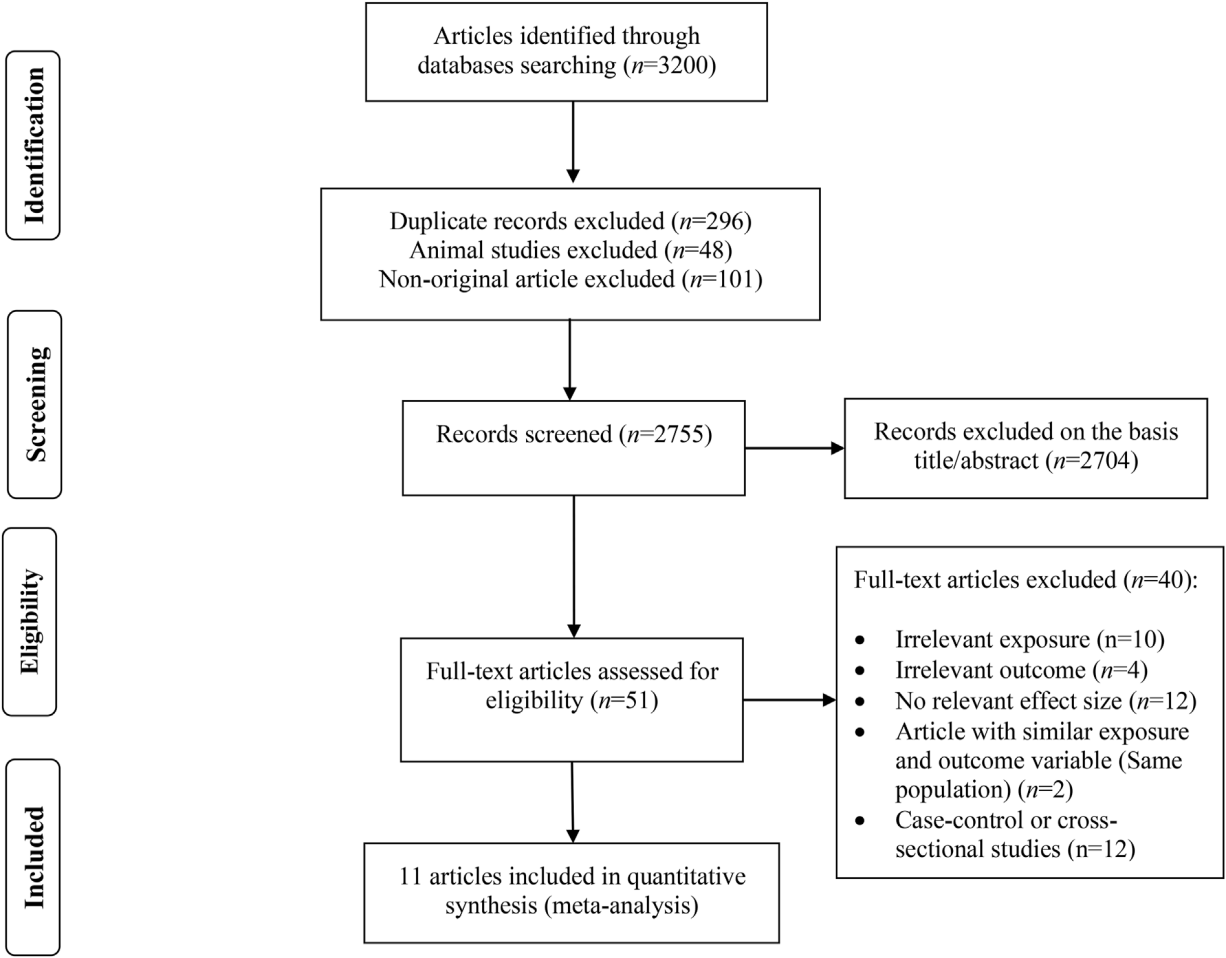
Flow diagram of study selection.

A total of 3200 records were identified from the initial search (1258 from PubMed, 760 from ISI Web of Science, 1161 from Embase, and 21 through other sources). Among these, 445 publications were duplicated, animal studies and nonoriginal articles. After title and abstract screening, 2704 irrelevant documents were removed. Consequently, by evaluating the full text, 40 articles were further excluded for various reasons, as shown in Supplemental Table 3. Finally, 11 prospective cohort studies with 8067 cases and 4,302,554 participants were considered eligible to be included in the current study [14,[35], [36], [37], [38], [39], [40], [41], [42], [43], [44]]. Figure 1 describes the diagram of study selection.

### Characteristics of studies

All population-based cohort studies were carried out in the general adult population. These cohort studies were dated from 2010 [35] to 2022 [44]. The follow-up period was between 1 and 27 y. Three studies were from the United States [36,37,39], and 8 were from the European regions [14,35,38,[40], [41], [42], [43], [44]]. Two cohorts were performed exclusively among women [35,39], whereas other investigations were conducted on both genders [14,[36], [37], [38],[40], [41], [42], [43], [44]]. For determining dietary animal protein sources intake, 9 cohorts used the FFQ [14,36,37,[39], [40], [41], [42], [43], [44]] and other studies used dietary habits [35,38]. We illustrate the characteristics of primary studies in Supplemental Table 4.

Nine cohort studies were judged at moderate risk of bias [14,[37], [38], [39], [40], [41], [42], [43], [44]], whereas 2 studies had a serious risk of bias [35,36] based on the Risk Of Bias In Non-randomized Studies-of Interventions tool. The existence of a potential risk of bias can be attributed to the possibility of residual confounding or insufficient information about the selection of participants (Supplemental Table 2).

### Findings from the Meta-Analysis

#### Total protein

**FIGURE 2:**
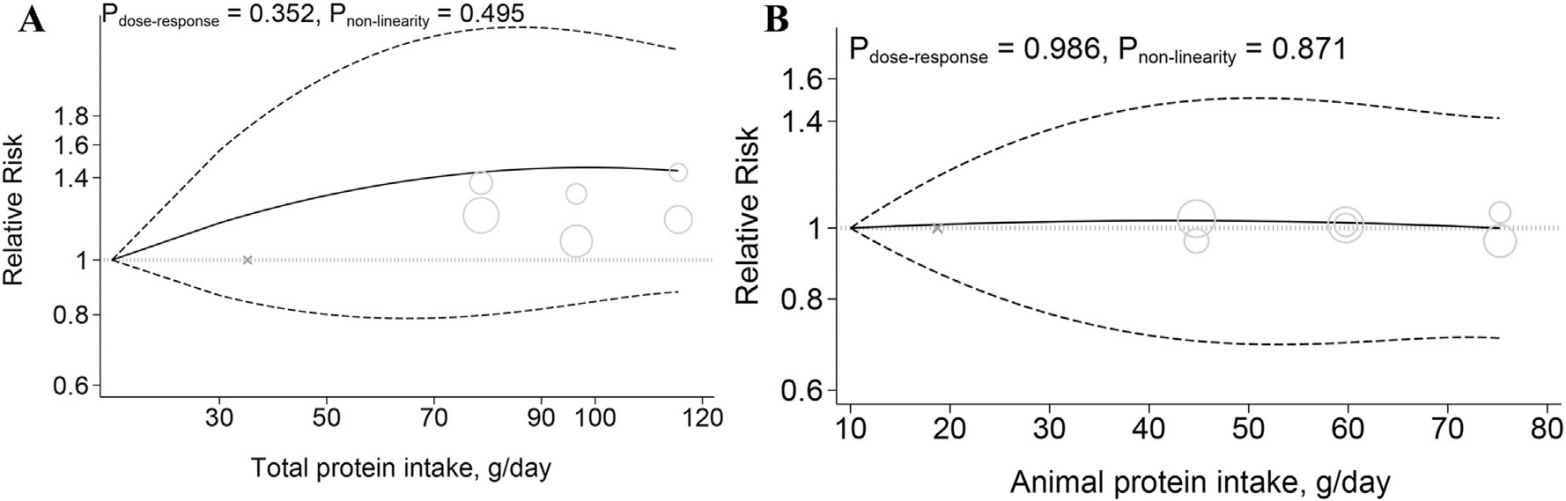
Dose-response associations of dietary protein intake and risk of inflammatory bowel diseases. (A) Total protein and (B) animal protein in random-effects models. Solid lines represent the RR of the association between dietary protein intake and inflammatory bowel disease and dashed lines represent 95% CI.

Four cohort studies (980 cases, 594,069 participants) examined the association between dietary total protein intake and risk of IBD [35,37,38,44]. The higher category of total protein intake was not associated with the risk of IBD (RR: 1.22; 95% CI: 0.88, 1.69; *n* = 4), CD (RR: 1.39; 95% CI: 0.80, 2.42; *n* = 3), and UC (RR: 1.20; 95% CI: 0.65, 2.21; *n* = 3) (Table 1). Moreover, results from a linear dose-response meta-analysis indicated that each additional 100 g of total protein per d was not associated with an increased risk of IBD (RR: 1.30; 95% CI: 0.87, 1.94; Table 1). There was no evidence of departure from linearity (Figure 2A).

**TABLE 1:** Total protein, animal protein, and animal protein sources with risk of inflammatory bowel diseases

	Pairwise meta-analysis (highest vs. lowest category meta-analysis)						Dose-response meta-analysis					
	Studies (*n*)	RR (95% CI)	*P* value	*I*^2^ (%)	*P*_heterogeneity_	Certainty of evidence	Dose, unit (g/)	Studies (*n*)	RR (95% CI)	*P* value	*I*^2^ (%)	*P*_heterogeneity_
Inflammatory bowel diseases												
Total protein	4	1.22 (0.88, 1.69)	0.225	46.3	0.097	⊕⊕◯◯ Low	100	2	1.30 (0.87, 1.94)	0.207	0.0	0.561
Animal protein	3	1.23 (0.81, 1.86)	0.341	62.5	0.031	⊕◯◯◯ Very low	100	2	0.98 (0.59, 1.64)	0.938	0.0	0.808
Red meat	6	1.10 (0.97, 1.25)	0.136	55.6	0.013	⊕⊕◯◯ Low	100	3	1.34 (0.96, 1.86)	0.085	33.4	0.223
Processed meat	5	1.09 (0.94, 1.26)	0.277	61.04	0.009	⊕◯◯◯ Very low	–	–	–	–	–	–
Poultry	2	1.18 (0.88, 1.59)	0.266	45.4	0.160	⊕◯◯◯ Very low	100	2	1.74 (0.49, 6.13)	0.392	51.9	0.125
Fish	5	1.03 (0.92, 1.15)	0.605	25.4	0.218	⊕⊕◯◯ Low	15	5	0.99 (0.88, 1.11)	0.809	49.8	0.093
Total meat	4	1.24 (0.90, 1.70)	0.182	66.8	0.029	⊕⊕◯◯ Low	100	3	1.38 (1.13, 1.68)	0.001	0.0	0.714
Dairy	7	0.81 (0.72, 0.90)	<0.001	36.2	0.101	⊕⊕⊕◯ Moderate	200	6	0.97 (092, 1.01)	0.173	0.0	0.627
Egg	3	0.92 (0.81, 1.04)	0.181	0.0	0.975	⊕⊕◯◯ Low	50	3	0.99 (0.65, 1.52)	0.968	0.0	0.912
Crohn disease												
Total protein	3	1.39 (0.80, 2.42)	0.241	27.6	0.251	–	–	–	–	–	–	–
Animal protein	3	1.34 (0.86, 2.10)	0.194	4.1	0.352	–	–	–	–	–	–	–
Red meat	5	1.02 (0.82, 1.28)	0.858	66.5	0.018	–	–	–	–	–	–	–
Processed meat	5	1.01 (0.78, 1.30)	0.943	65.9	0.019	–	–	–	–	–	–	–
Poultry	1	1.42 (0.87, 2.33)	0.165	–	–	–	–	–	–	–	–	–
Fish	4	0.92 (0.73, 1.15)	0.451	43.4	0.151	–	–	–	–	–	–	–
Total meat	1	1.28 (0.76, 2.16)	0.354	–	–	–	–	–	–	–	–	–
Dairy	5	0.69 (0.56, 0.86)	0.001	50.2	0.090	–	–	–	–	–	–	–
Egg	2	0.94 (0.78, 1.12)	0.489	0.0	0.583	–	–	–	–	–	–	–
Ulcerative colitis												
Total protein	3	1.20 (0.65, 2.21)	0.555	65.9	0.053		–	–	–	–	–	–
Animal protein	3	1.20 (0.63, 2.30)	0.583	74.2	0.021	–	–	–	–	–	–	–
Red meat	5	1.16 (0.96, 1.40)	0.123	59.0	0.045	–	–	–	–	–	–	–
Processed meat	5	1.16 (0.99, 1.37)	0.067	32.0	0.208	–	–	–	–	–	–	–
Poultry	1	0.92 (0.67, 1.26)	0.605	–	–	–	–	–	–	–	–	–
Fish	4	1.07 (0.95, 1.20)	0.266	0.0	0.856	–	–	–	–	–	–	–
Total meat	1	1.40 (0.99, 1.98)	0.057	–	–	–	–	–	–	–	–	–
Dairy	5	0.84 (0.75, 0.94)	0.003	0.0	0.988	–	–	–	–	–	–	–
Egg	2	0.90 (0.76, 1.07)	0.231	0.0	0.824	–	–	–	–	–	–	–

#### Animal protein

Three studies with 845 cases (total *n* = 565,974) were included in this association [35,37,44]. The summary RR of IBD, CD, and UC for the highest compared with the lowest category of animal protein intake was 1.23 (95% CI: 0.81, 1.86; *n* = 3), 1.34 (95% CI: 0.86, 2.10; *n* = 3), and 1.20 (95% CI: 0.63, 2.30; *n* = 3), respectively (Table 1). Furthermore, there was no linear association between dietary animal protein intake and risk of IBD (RR: 0.98; 95% CI: 0.59, 1.64; Table 1) with no evidence of departure from linearity (Figure 2B).

#### Red meat intake

Six cohort studies involving 7383 cases of IBD among 3,666,182 participants were eligible in the analysis of dietary red meat intake [36,39,40,[42], [43], [44]]. The results of the highest compared with lowest analysis revealed that there was no significant association between dietary red meat intake and the risk of IBD (RR: 1.10; 95% CI: 0.97, 1.25; *n* = 6), CD (RR: 1.02; 95% CI: 0.82, 1.28; *n* = 5), and UC (RR: 1.16; 95% CI: 0.96, 1.40; *n* = 5). In the subgroup analyses, geographic locations and those that controlled for physical activity were identified as potential sources of heterogeneity (Supplemental Table 5).

**FIGURE 3:**
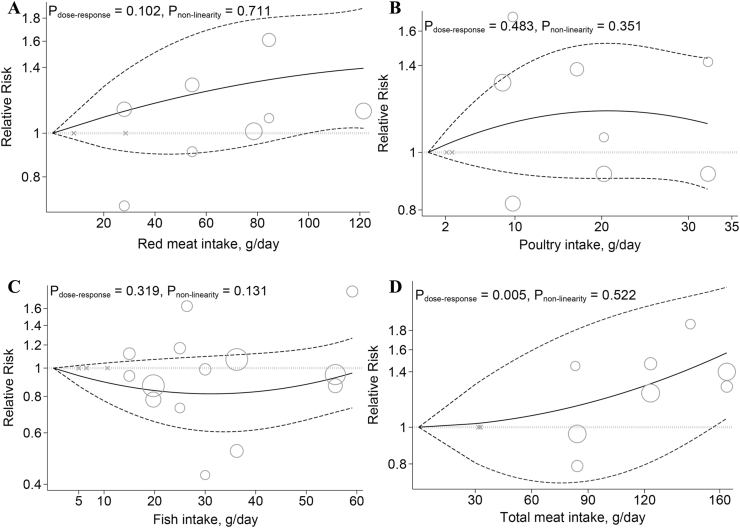
Dose-response associations of dietary meat intake and risk of inflammatory bowel diseases. (A) Red meat, (B) poultry, (C) fish, and (D) total meat in random-effects models. Solid lines represent the RR of the association between dietary meat intake and inflammatory bowel disease and dashed lines represent 95% CI.

In addition, no significant association was found between an increment of 100 g of red meat intake per d and the risk of IBD (RR: 1.34; 95% CI: 0.96, 1.86; Table 1). We observed no evidence of departure from linearity (Figure 3A).

#### Processed meat

Five cohort studies (total *n* = 3,583,035) with 6824 cases were considered in the analysis of the highest and lowest categories of processed meat intake and risk of IBD [36,39,[42], [43], [44]]. The highest compared with lowest category of processed meat intake was not associated with risk of IBD (RR: 1.09; 95% CI: 0.94, 1.26; *n* = 5), CD (RR: 1.01; 95% CI: 0.78, 1.30; *n* = 5), and UC (RR: 1.16; 95% CI: 0.99, 1.37; *n* = 5) (Table 1).

#### Poultry intake

Two studies composed of 529,677 subjects and 1071 IBD cases were analyzed for the relation between dietary poultry intake and the risk of IBD [43,44]. The highest compared with the lowest category of poultry intake was not associated with risk of IBD (RR: 1.18; 95% CI: 0.88, 1.59; *n* = 2), CD (RR: 1.42; 95% CI: 0.87, 2.33; *n* = 1), and UC (RR: 0.92; 95% CI: 0.67, 1.26; *n* = 1) (Table 1).

A linear dose-response investigated that each 100 g/d increase in dietary poultry intake was not associated with the risk of IBD (RR: 1.74; 95% CI: 0.49, 6.13; Table 1). There was no evidence of departure from linearity between dietary poultry intake and risk of IBD (Figure 3B).

#### Fish

Five cohort studies (total *n* = 660,429) with 4757 cases evaluated the relation between dietary fish intake and risk of IBD [35,37,40,42,44]. The pooled analysis of highest compared with lowest analysis revealed that dietary fish intake was not significantly associated with a lower risk of IBD (RR: 1.03; 95% CI: 0.92, 1.15; *n* = 5), CD (RR: 0.92; 95% CI: 0.73, 1.15; *n* = 4), and UC (RR: 1.07; 95% CI: 0.95, 1.20; *n* = 4) (Table 1).

Each 15 g/d increment in dietary fish intake was not associated with the risk of IBD (RR: 0.99; 95% CI: 0.88, 1.11; Table 1). There was no evidence of departure from linearity between dietary fish intake and risk of IBD (Figure 3C).

#### Total meat

Three prospective cohort studies with 1214 cases of IBD among a total of 535,738 subjects were included in the total meat analyses [35,41,44]. Higher dietary total meat intake was not significantly associated with the risk of IBD (RR: 1.24; 95% CI: 0.90, 1.70; *n* = 4), CD (RR: 1.28; 95% CI: 0.76, 2.16; *n* = 1), and UC (RR: 1.40; 95% CI: 0.99, 1.98; *n* = 1) (Table 1).

Findings indicated that each 100 g/d increment in dietary total meat consumption was associated with a 38% greater risk of IBD (RR: 1.38, 95% CI: 1.13, 1.68, Table 1). Moreover, a positive linear association was observed between total meat intake and the risk of IBD (Figure 3D).

#### Dairy

Seven cohort studies (7232 cases, 1,097,040 participants) were included in the analysis of dairy intake [14,35,36,40,[42], [43], [44]]. Comparing the highest and lowest categories for dietary dairy intake was significantly associated with a lower risk of IBD (RR: 0.81; 95% CI: 0.72, 0.90; *n* = 7), CD (RR: 0.69; 95% CI: 0.56, 0.86; *n* = 5), and UC (RR: 0.84; 95% CI: 0.75, 0.94; *n* = 5) (Table 1). However, subgroup analyses based on the case ascertainment method revealed that dairy consumption was significantly associated with a reduced risk of IBD among studies using medical records (RR = 0.77; 95% CI: 0.68, 0.88) as opposed to self-report and ICD questionnaires. In addition, a significant inverse association persisted across studies without adjustment for EI, alcohol, and BMI. Also, a significant inverse association persisted even after adjustment for sex (Supplemental Table 6).

**FIGURE 4:**
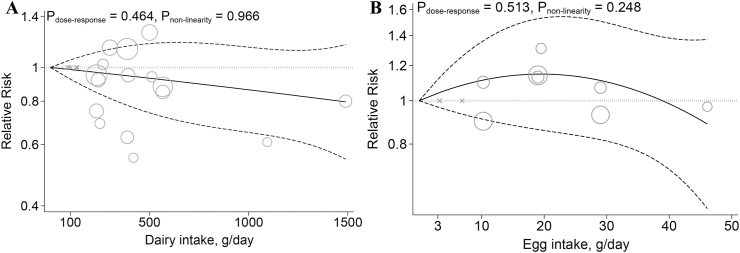
Dose-response associations of dietary animal protein sources intake and risk of inflammatory bowel diseases. (A) Dairy and (B) egg in random-effects models. Solid lines represent the RR of the association between dietary animal protein sources intake and inflammatory bowel disease and dashed lines represent 95% CI.

The dose-response analysis revealed that each additional 200 g of dairy consumption per day was not significantly associated with a lower risk of IBD (RR: 0.97; 95% CI: 0.92, 1.01; Table 1). There was no evidence of departure from linearity (Figure 4A).

#### Egg

Three prospective cohorts, among 492,497 participants and 4025 cases, were analyzed in this relation [35,42,44]. The highest compared with the lowest category of egg consumption was not associated with risk of IBD (RR: 0.92; 95% CI: 0.81, 1.04; *n* = 4), CD (RR: 0.94; 95% CI: 0.78, 1.12; *n* = 2), and UC (RR: 0.90; 95% CI: 0.76, 1.07; *n* = 2) (Table 1).

No linear association between dietary egg intake and risk of IBD was found (RR: 0.99; 95% CI: 0.65, 1.52; Table 1). We observed no evidence of departure from linearity between dietary egg intake and the risk of IBD (Figure 4B).

### Publication Bias and Sensitivity Analyses

There was no evidence of publication bias from Begg’s and Egger’s tests. The sensitivity analysis results for the risk of IBD revealed that the exclusion of studies by Khalili et al. [39], Robin et al. [41], and Dong et al. [44] for red meat, total meat, and poultry altered the primary analyses, respectively. Also, the exclusion of Cohen et al. [36] modified the overall impact of dietary intake of processed meat and the risk of IBD and UC. However, the remaining effect sizes were unaffected by any cohort studies.

### Grading the Evidence

The certainty of the evidence for the association between total dietary protein, as well as animal protein, and its sources intake and IBD incidence was rated using the GRADE method, and its details are shown in Supplemental Table 7. The certainty of the evidence was rated moderate to very low because of degradation of inconsistency, imprecision, and risk of bias.

## Discussion

Because of the increase in the prevalence of IBD in the world, the research on the effective factors in the occurrence and exacerbation of the symptoms of this disease has increased significantly, and one of the important factors is the diet of the patients [45,46]. In the present study, we investigated the effect of protein consumption both in terms of total protein intake and also by the type of protein on the risk of IBD occurrences, and results showed that each 100 g/d increment in dietary total meat consumption was associated with a 38% greater risk of IBD. Also, the results of our study showed a protective role of dairy intake against the risk of IBD. However, we did not find any other significant association between other types of protein intake with the risk of IBD.

Considerable intakes of protein, carbohydrates, and fat are the main features of the Western dietary pattern, which has become more and more popular throughout Asia over the past few decades, and surprisingly, the incidence of IBD, especially UC, has increased significantly in this area during this period of time [47,48]. In line with our findings, in 2015, Ge et al. [49] showed that meat consumers had a significantly higher risk of IBD than those who reported no or low meat consumption. Also, Zhou et al. [11] in a meta-analysis study showed a nonsignificant association between a total dietary protein with the risk of IBD. However, in the subgroup analysis, they found a positive relationship between dietary protein intake and IBD risk among Asian populations but not among European populations. However, their analysis mainly included case-control studies, cohort studies were not included in the analysis, and they only examined total protein consumption without considering the type of protein and its source.

Several mechanisms have been proposed to explain the relation between meat intake, especially red meat, and the risk of IBD. It seems that the effect of the high consumption of meats in increasing the risk of IBD is not related to the type of protein contained in it but rather to other reasons. One of the proposed mechanisms is the role of metabolites produced during cooking meat at high temperatures and the inappropriate effect of these compounds on the digestive tract [50]. The classes of compounds formed during high-temperature or open-flame cooking include heterocyclic amines and polycyclic aromatic hydrocarbons. The heterocyclic amine in meat process 2-amino-3-dimethylimidazo [4,5-f] quinoxaline, 2-amino-3,8-dimethylimidazo [4,5-f] quinoxaline, 2-amino-3,4,8-trimethylimidazo [4,5-f] quinoxaline, and 2-amino-1 methyl-6-phenylimidazo [4,5-b] pyridine. The most prominent polycyclic aromatic hydrocarbon in meat is benzo(a)pyrene. Previous studies have shown the adverse effect of these metabolites in the occurrence of gastrointestinal cancers, such as colon cancer [51]. Also, the presence of compounds, such as the high amount of iron in meat foods, N-nitroso compounds, and SFAs content have been suggested in the interpretation of this result [35,52]. Moreover, in some studies, it has been reported that the type of dietary protein can affect the risk of inflammatory diseases by influencing the intestinal microbial profile. For example, it has been shown that consumption of whey and pea protein extract has been reported to increase gut-commensal *Bifidobacterium* and *Lactobacillus*, whereas whey additionally decreases the pathogenic *Bacteroides fragilis* and *Clostridium perfringens* [53,54]. Furthermore, several microbial genera promoted by the intake of red meat have also been associated with increased levels of trimethylamine-N-oxide [55], which in recent years has been considered a new factor in diagnosing and investigating the progress of IBD [56].

We did not find any significant association between red or processed meat consumption and with risk of IBD, CD, or UC. In the subgroup analysis, there was a significant association between red meat intake and the risk of IBD in studies conducted in Europe. This difference in results can be caused by differences in cooking methods, genetic factors, amount of meat consumption, confounding factors, and other environmental factors. We did not find any significant correlation between animal protein intake and the risk of IBD incidence. Some previous studies have reported that the consumption of animal proteins, especially red meat, increases the risk of IBD [35,57]. They suggested some biologic plausibility for a positive association between animal protein intake and IBD. For example, a variable proportion of heme and amino acids, contained in animal proteins, are not absorbed by the small bowel and reach the colonic lumen, where they are metabolized by the microflora. This produces a number of byproducts, some of which may be harmful to the colon, such as hydrogen sulfide, phenolic compounds, amines, and ammonia. For example, it has been proposed that sulfide can change the colonocyte’s cell membrane structure in the presence of nitric oxide generated by anaerobic bacteria, resulting in the impairment of barrier function and the inflammatory cascade seen in UC [58]. However, these contradictions in the results of some previous studies can be because of reasons, such as nested case-control design, intake of protein considered as a percentage of total energy, including alcohol, heterogeneity between centers, and dietary questionnaires, examining the association between dietary protein and the risk of IBD in one gender (total population only men or women).

In the present study, we found an inverse association between dairy intake and the risk of IBD, CD, and UC. However, this significant association was observed in studies that did not control for alcohol intake or EI. Adjusting for these variables would likely attenuate the association, as studies in which all confounding factors, etc, have been included have not seen a significant association. In studies in which all confounding factors such as alcohol intake, BMI, and EI were adjusted, no significant relationship was found between the consumption of dairy products and the risk of IBD. In line with our findings, in some previous studies, there was not any significant correlation between total dairy intake and the risk of IBD. But when researchers performed subgroup analysis by dairy product type, there was an inverse association between milk intake and odds of developing CD compared with nonconsumers [14].

Evidence for a causal association is the plausible biologic mechanisms, large effect sizes, and temporality of data collection. According to several studies, milk products may assist in decreasing intestinal inflammation, perhaps by directly reducing inflammatory processes or influencing the gut flora [[59], [60], [61], [62]]. The beneficial effects of milk on intestinal inflammation can be due to the anti-inflammatory properties of vitamin D [63], as well as the positive role of milk in the production of butyrate in the colon [64], a crucial energy supplier for the colon [65]. However, the results of the studies are contradictory. In a previous animal model study, it was reported that milk-derived SFAs administration led to the development of colitis by affecting the microbial population and types and especially changing the composition of bile acids [66]. This suggests that various dairy constituents, such as fats or proteins, may have varying impacts, highlighting the fact that the precise mechanisms underlying these relationships are yet unknown. The results of a study on the people of Iceland showed that 60% of patients with IBD decreased their intake of dairy products and >87% of them believed that their diet had an impact on their gastrointestinal symptoms [67].

Overall, our results did not show any significant correlation or a dose-response association between other dietary protein sources, such as fish, egg, poultry, or processed meat, with the risk of IBD, CD, or UC. To the best of our knowledge, the present study was the first systematic review and dose-response meta-analysis that evaluated the association between dietary protein intake and the risk of IBD. The strengths of this study include the dose-response analyses and rigorous evaluation of the methodological quality of the included studies, the use of the grading system for reporting the certainty of the evidence, and including only cohort studies that had a high sample size and power. As mentioned before, in the highest compared with lowest analyses, the higher protein intake category (tertiles, quartiles, or quintiles) in comparison with the lowest protein intake category was compiled for analysis. This is a routine method; however, it is relatively restrictive because it cannot determine the optimum dose required for decision making. Thus, we further evaluated our data with dose-response analysis, which could help us better understand whether the risk of IBD was affected by increased intake of protein. However, this study had some limitations that should be considered in the interpretation of the data. First, retrospective assessments of dietary or other factors may have resulted in recall bias. Second, high heterogeneity was found in some of the investigated factors. Third, some of the evaluated studies had moderate and low quality from a methodological point of view.

In conclusion, the results showed no significant relationship between the consumption of most dietary protein sources and the incidence of IBD. Among the dietary sources of proteins, the risk of IBD increased only with increasing total meat intake, and the consumption of protein from dairy products was found to be a protective factor against the IBD risk. More studies, especially intervention trials, are needed to confirm the results of this study.

## Funding

Supported by Tehran University of Medical Sciences’ Students’ Scientific Research Center (SSRC) (code: 1401-4-125-64978).

## Author disclosures

The authors report no conflicts of interest.

## Appendix A Supplementary data

Multimdia component 1 Multimdia component 1
