# Supplementary material for: The Association between Total Protein, Animal Protein, and Animal Protein Sources with Risk of Inflammatory Bowel Diseases: A Systematic Review and Meta-Analysis of Cohort Studies
Source: Adv Nutr. 2023 May 14;14(4):752–61. doi: 10.1016/j.advnut.2023.05.008 (PMC10334156; doi:10.1016/j.advnut.2023.05.008)
Supplement: Multimdia component 1 [file mmc1.docx]

**The association between total protein, animal protein, and animal protein sources with risk of** **inflammatory bowel diseases: a systematic review and meta-analysis of cohort studies**

Talebi et al.

Online Supplementary Material

Supporting Materials including Supplementary Tables 1-7, and Supplementary references

| **Supplementary Table 1.** Search strategies including the key terms and the queries for each database | |
| --- | --- |
| **Database**  **3/28/2023**  **March 28, 2023** | **key terms and the queries** |
| PubMed  N=1285 | #1 "Dairy Products"[MeSH Terms] OR "Milk"[MeSH Terms] OR "Yogurt"[MeSH Terms] OR "Cheese"[MeSH Terms] OR "Fishes"[MeSH Terms] OR "Seafood"[MeSH Terms] OR "Meat"[MeSH Terms] OR "Poultry"[MeSH Terms] OR "food*"[All Fields] OR "egg"[All Fields] OR "dairy"[All Fields] OR "dairies"[All Fields] OR "Milk"[All Fields] OR "Yogurt"[All Fields] OR "Cheese"[All Fields] OR "fish"[All Fields] OR "Seafood"[All Fields] OR "Meat"[All Fields] OR "processed meat"[All Fields] OR "Poultry"[All Fields] OR "Fast Foods"[MeSH Terms] OR "Meat Proteins"[MeSH Terms] OR "Red Meat"[MeSH Terms] OR "Pork Meat"[MeSH Terms] OR "fast food"[All Fields] OR "convenience food"[All Fields] OR "ready to eat foods"[All Fields] OR "ready prepared foods"[All Fields] OR "meat protein"[All Fields] OR "Red Meat"[All Fields] OR "pork"[All Fields] OR "beef"[All Fields] OR "processed red meat"[All Fields] OR "Animal proteins"[All Fields] OR "Dietary Proteins"[All Fields] OR "Dietary Proteins"[MeSH Terms] OR "protein intake"[All Fields] OR "total protein"[All Fields]  #2 ("Inflammatory Bowel Diseases"[MeSH Terms] OR "Inflammatory Bowel Diseases"[Title/Abstract] OR "Inflammatory Bowel Disease"[Title/Abstract] OR "IBD"[Title/Abstract] OR "colitis, ulcerative"[MeSH Terms] OR "colitis ulcerative"[Title/Abstract] OR "Idiopathic Proctocolitis"[Title/Abstract] OR "Ulcerative Colitis"[Title/Abstract] OR "Colitis Gravis"[Title/Abstract] OR "Crohn Disease"[MeSH Terms] OR "Crohn Disease"[Title/Abstract] OR "Crohn's Enteritis"[Title/Abstract] OR "Regional Enteritis"[Title/Abstract] OR "Crohn's Disease"[Title/Abstract] OR "Crohns Disease"[Title/Abstract] OR "Granulomatous Enteritis"[Title/Abstract] OR "Ileocolitis"[Title/Abstract] OR "Granulomatous Colitis"[Title/Abstract] OR "Terminal Ileitis"[Title/Abstract] OR "Regional Ileitis"[Title/Abstract])  #3 "prospective"[All Fields] OR "prospectively"[All Fields] OR "longitudinal"[All Fields] OR "cohort"[All Fields] OR "cohorts"[All Fields] OR "observation"[All Fields] OR "observational"[All Fields] OR "follow up"[All Fields] OR "nested"[All Fields] OR "case control"[All Fields] OR "case controls"[All Fields] OR "nested case control"[All Fields] OR "Cohort Studies"[MeSH Terms] OR "Cohort Studies"[MeSH Terms] OR "Case-Control Studies"[MeSH Terms] OR "Longitudinal Studies"[MeSH Terms] OR "Prospective Studies"[MeSH Terms] OR "Observational Study"[Publication Type] OR "Cross-Sectional Studies"[All Fields] OR "Cross-Sectional"[All Fields] OR "Cross-Section"[All Fields] OR "Cross-Sectional Studies"[MeSH Terms]  #4 #1 AND #2 |
| Web of Science (ISI)  N=760 | #1 "Dairy Products" (Topic) or "Milk" (Topic) or "Cheese" (Topic) or "Yogurt" (Topic) or "dairy" (Topic) or "dairies" (Topic) or "egg" (Topic) or "food*" (Topic) or "fish" (Topic) or "Seafood" (Topic) or "Meat" (Topic) or "processed meat" (Topic) or "Poultry" (Topic) or "Fast Foods" (Topic) or "Meat Proteins" (Topic) or "Red Meat" (Topic) or "Pork Meat" (Topic) or "convenience food" (Topic) or "ready to eat foods" (Topic) or "ready prepared foods" (Topic) or "meat protein" (Topic) or "processed red meat" (Topic) or "beef" (Topic) or "Animal proteins" (Topic) or "Dietary Proteins" (Topic) or "protein intake" (Topic) or “total protein” (Topic)  #2 "Inflammatory Bowel Diseases" (Topic) or "Inflammatory Bowel Disease" (Topic) or "IBD" (Topic) or "colitis, ulcerative" (Topic) or "colitis ulcerative" (Topic) or "Idiopathic Proctocolitis" (Topic) or "Ulcerative Colitis" (Topic) or "Colitis Gravis" (Topic) or "Crohn Disease" (Topic) or "Crohn's Enteritis" (Topic) or "Regional Enteritis" (Topic) or "Crohn's Disease" (Topic) or "Crohns Disease" (Topic) or "Granulomatous Enteritis" (Topic) or "Ileocolitis" (Topic) or "Granulomatous Colitis" (Topic) or "Terminal Ileitis" (Topic) or "Regional Ileitis" (Topic)  #3 "prospective" (Topic) or "prospectively” (Topic) or "longitudinal" (Topic) or "cohort" (Topic) or "cohorts" (Topic) or "observation" (Topic) or "observational" (Topic) or "follow up" (Topic) or "nested" (Topic) or "nested case control" (Topic) or "Cohort Studies" (Topic) or "Longitudinal Studies" (Topic) or "Prospective Studies" (Topic) or "Observational Study" (Topic) or "Case-Control Studies" (Topic) or "case controls" (Topic) or "cross section" (Topic) or "cross-sectional Studies" (Topic) or "cross-sectional" (Topic)  #4 #1 AND #2 AND #3 |
| Embase  (n=1161)  28 March 2023 | #1 'dairy products':ab,ti OR 'milk':ab,ti OR 'cheese':ab,ti OR 'yogurt':ab,ti OR 'dairy':ab,ti OR 'dairies':ab,ti OR 'egg':ab,ti OR 'food*':ab,ti OR 'fish':ab,ti OR 'seafood':ab,ti OR 'meat':ab,ti OR 'processed meat':ab,ti OR 'poultry':ab,ti OR 'fast foods':ab,ti OR 'meat proteins':ab,ti OR 'red meat':ab,ti OR 'pork meat':ab,ti OR 'convenience food':ab,ti OR 'ready to eat foods':ab,ti OR 'ready prepared foods':ab,ti OR 'meat protein':ab,ti OR 'processed red meat':ab,ti OR 'beef':ab,ti OR 'animal proteins':ab,ti OR 'dietary proteins':ab,ti OR 'protein intake':ab,ti OR 'total protein':ab,ti  #2 'inflammatory bowel diseases':ab,ti OR 'inflammatory bowel disease':ab,ti OR 'ibd':ab,ti OR 'colitis, ulcerative':ab,ti OR 'colitis ulcerative':ab,ti OR 'idiopathic proctocolitis':ab,ti OR 'ulcerative colitis':ab,ti OR 'colitis gravis':ab,ti OR 'crohn disease':ab,ti OR 'crohn enteritis':ab,ti OR 'regional enteritis':ab,ti OR 'crohns disease':ab,ti OR 'granulomatous enteritis':ab,ti OR 'ileocolitis':ab,ti OR 'granulomatous colitis':ab,ti OR 'terminal ileitis':ab,ti OR 'regional ileitis':ab,ti  'prospective':ab,ti OR 'prospectively':ab,ti OR 'longitudinal':ab,ti OR 'cohort':ab,ti OR 'cohorts':ab,ti OR 'observation':ab,ti OR 'observational':ab,ti OR 'follow up':ab,ti OR 'nested':ab,ti OR 'nested case control':ab,ti OR 'cohort studies':ab,ti OR 'longitudinal studies':ab,ti OR 'prospective studies':ab,ti OR 'observational study':ab,ti OR 'case-control studies':ab,ti OR 'case controls':ab,ti OR 'cross section':ab,ti OR 'cross-sectional studies':ab,ti OR 'cross-sectional':ab,ti  #4 #1 AND #2 AND #3 |

| **Table S2**: ROBINS-I judgement for each domain and overall | | | | | | | | |
| --- | --- | --- | --- | --- | --- | --- | --- | --- |
| **Author (Country, year)** | **Bias of confounding** | **Bias in selection of participants into the study** | **Bias due to exposure assessment** | **Bias due to misclassification during follow-up** | **Bias of missing data** | **Bias in measurement of outcomes** | **Bias in selection of the reported result** | **Overall judgement** |
| Rubin et al (Denmark, 2020) (1) | Moderate | Low | Moderate | Moderate | Low | Low | Low | **Moderate** |
| Dong et al, (Eight European countries, 2022) (2) | Moderate | Low | Moderate | Moderate | Moderate | Moderate | Low | **Moderate** |
| Julià et al. (Spain, 2021) (3) | Moderate | Low | Moderate | Low | Low | Low | Low | **Moderate** |
| Ananthakrishnan et al. (USA, 2015) (4) | Moderate | Low | Moderate | Moderate | Moderate | Moderate | Low | **Moderate** |
| Narula et al. (21countries, 2021) (5) | Moderate | Low | Moderate | Low | Moderate | Moderate | Low | **Moderate** |
| Cohen et al. (USA, 2013) (6) | Serious | Low | Moderate | Moderate | Low | Low | Low | **Serious** |
| Khalili et al. (Sweden, 2020) (7) | Moderate | Low | Moderate | Moderate | Moderate | Low | Low | **Moderate** |
| Khalili et al. (USA, 2017) (8) | Moderate | Low | Moderate | Moderate | Moderate | Moderate | Low | **Moderate** |
| Larsson et al. (Sweden, 2016) (9) | Moderate | Low | Moderate | Low | Low | Low | Low | **Moderate** |
| Opstelten et al. (European (12 centers), 2016) (10) | Moderate | Low | Moderate | Moderate | Low | Low | Low | **Moderate** |
| Jantchou et al. (France, 2010) (11) | Serious | Low | Moderate | Moderate | Low | Low | Low | **Serious** |

| **Table S3**: Reason for exclusion of retrieved articles | |
| --- | --- |
| References | Reason for exclusion |
| 1. Buscail C, Sabate JM, Bouchoucha M, Torres MJ, Allès B, Hercberg S, Benamouzig R, Julia C. Association between self-reported vegetarian diet and the irritable bowel syndrome in the French NutriNet cohort. PLoS One. 2017 Aug 25;12(8):e0183039. 2. Chen H, Fu T, Dan L, Chen X, Sun Y, Chen J, Wang X, Hesketh T. Meat consumption and all-cause mortality in 5763 patients with inflammatory bowel disease: A retrospective cohort study. EClinicalMedicine. 2022 May 1;47:101406. 3. Andersen V, Holst R, Kopp TI, Tjønneland A, Vogel U. Interactions between diet, lifestyle and IL10, IL1B, and PTGS2/COX-2 gene polymorphisms in relation to risk of colorectal cancer in a prospective Danish case-cohort study. PLoS One. 2013 Oct 23;8(10):e78366. 4. Overgaard SH, Sørensen SB, Munk HL, Nexøe AB, Glerup H, Henriksen RH, Guldmann T, Pedersen N, Saboori S, Hvid L, Dahlerup JF. Impact of fibre and red/processed meat intake on treatment outcomes among patients with chronic inflammatory diseases initiating biological therapy: A prospective cohort study. Frontiers in nutrition. 2022:2421. | Not relevant outcome |
| 1. Naqvi SA, Taylor LM, Panaccione R, Ghosh S, Barkema HW, Hotte N, Shommu N, Kaur S, Reimer RA, Madsen KL, Raman M. Dietary patterns, food groups and nutrients in Crohn’s disease: associations with gut and systemic inflammation. Scientific Reports. 2021 Jan 18;11(1):1-9. 2. Bueno-Hernández N, Núñez-Aldana M, Ascaño-Gutierrez I, Yamamoto-Furusho JK. Evaluation of diet pattern related to the symptoms of mexican patients with Ulcerative Colitis (UC): through the validity of a questionnaire. Nutrition journal. 2015 Dec;14(1):1-8. 3. Guida L, Di Giorgio FM, Busacca A, Carrozza L, Ciminnisi S, Almasio PL, Di Marco V, Cappello M. Perception of the role of food and dietary modifications in patients with inflammatory bowel disease: Impact on lifestyle. Nutrients. 2021 Feb 26;13(3):759. 4. Vagianos K, Clara I, Carr R, Graff LA, Walker JR, Targownik LE, Lix LM, Rogala L, Miller N, Bernstein CN. What are adults with inflammatory bowel disease (IBD) eating? A closer look at the dietary habits of a population‐based Canadian IBD cohort. Journal of Parenteral and Enteral Nutrition. 2016 Mar;40(3):405-11. 5. de Graaf MC, Spooren CE, Hendrix EM, Hesselink MA, Feskens EJ, Smolinska A, Keszthelyi D, Pierik MJ, Mujagic Z, Jonkers DM. Diet Quality and Dietary Inflammatory Index in Dutch Inflammatory Bowel Disease and Irritable Bowel Syndrome Patients. Nutrients. 2022 May 6;14(9):1945. 6. Keshteli AH, van den Brand FF, Madsen KL, Mandal R, Valcheva R, Kroeker KI, Han B, Bell RC, Cole J, Hoevers T, Wishart DS. Dietary and metabolomic determinants of relapse in ulcerative colitis patients: a pilot prospective cohort study. World Journal of Gastroenterology. 2017 Jun 6;23(21):3890. 7. WENG Y, GAN H, LI X, HUANG Y, LI Z, DENG H, CHEN S, ZHOU Y, WANG L, HAN Y, TAN Y. Diet-microbiota-metabolite interaction networks reveal key players in inflammatory bowel disease. J. Dig. Dis. 2019. 8. Vagianos K, Shafer LA, Witges K, Targownik LE, Haviva C, Graff LA, Sexton KA, Lix LM, Sargent M, Bernstein CN. Association between change in inflammatory aspects of diet and change in IBD-related inflammation and symptoms over 1 year: the Manitoba Living With IBD study. Inflammatory Bowel Diseases. 2021 Feb;27(2):190-202. 9. Casanova MJ, Chaparro M, Molina B, Merino O, Batanero R, Dueñas-Sadornil C, Robledo P, Garcia-Albert AM, Gómez-Sánchez MB, Calvet X, Trallero MD. Prevalence of malnutrition and nutritional characteristics of patients with inflammatory bowel disease. Journal of Crohn's and Colitis. 2017 Dec 4;11(12):1430-9. 10. Peters V, Spooren CE, Pierik MJ, Weersma RK, van Dullemen HM, Festen EA, Visschedijk MC, Masclee AA, Hendrix EM, Almeida RJ, Perenboom CW. Dietary intake pattern is associated with occurrence of flares in IBD patients. Journal of Crohn's and Colitis. 2021 Aug;15(8):1305-15. 11. Hammer T, Lophaven SN, Nielsen KR, Petersen MS, Munkholm P, Weihe P, Burisch J, Lynge E. Dietary risk factors for inflammatory bowel diseases in a high-risk population: results from the Faroese IBD study. United European Gastroenterology Journal. 2019 Aug;7(7):924-32. 12. González-Delgado P, Muriel J, Jiménez T, Cameo JI, Palazón-Bru A, Fernández J. Food Protein–Induced Enterocolitis Syndrome in Adulthood: Clinical Characteristics, Prognosis, and Risk Factors. The Journal of Allergy and Clinical Immunology: In Practice. 2022 Sep 1;10(9):2397-403. | Without sufficient data |
| 1. Racine A, Carbonnel F, Chan SS, Hart AR, Bueno-de-Mesquita HB, Oldenburg B, Van Schaik FD, Tjønneland A, Olsen A, Dahm CC, Key T. Dietary patterns and risk of inflammatory bowel disease in Europe: results from the EPIC study. Inflammatory bowel diseases. 2016 Feb 1;22(2):345-54. 2. Ananthakrishnan AN, Khalili H, Konijeti GG, Higuchi LM, de Silva P, Fuchs CS, Willett WC, Richter JM, Chan AT. Long-term intake of dietary fat and risk of ulcerative colitis and Crohn's disease. Gut. 2014 May 1;63(5):776-84. 3. Ananthakrishnan AN, Khalili H, Konijeti GG, Higuchi LM, De Silva P, Korzenik JR, Fuchs CS, Willett WC, Richter JM, Chan AT. A prospective study of long-term intake of dietary fiber and risk of Crohn's disease and ulcerative colitis. Gastroenterology. 2013 Nov 1;145(5):970-7. 4. Brotherton CS, Martin CA, Long MD, Kappelman MD, Sandler RS. Avoidance of fiber is associated with greater risk of Crohn’s disease flare in a 6-month period. Clinical Gastroenterology and Hepatology. 2016 Aug 1;14(8):1130-6. 5. Lo CH, Lochhead P, Khalili H, Song M, Tabung FK, Burke KE, Richter JM, Giovannucci EL, Chan AT, Ananthakrishnan AN. Dietary inflammatory potential and risk of Crohn’s disease and ulcerative colitis. Gastroenterology. 2020 Sep 1;159(3):873-83. 6. Khalili H, Hakansson N, Chan SS, Ludvigsson JF, Olen O, Chan AT, Hart AR, Wolk A. No association between consumption of sweetened beverages and risk of later-onset Crohn’s disease or ulcerative colitis. Clinical Gastroenterology and Hepatology. 2019 Jan 1;17(1):123-9. 7. Mirmiran P, Moslehi N, Morshedzadeh N, Shivappa N, Hébert JR, Farsi F, Daryani NE. Does the inflammatory potential of diet affect disease activity in patients with inflammatory bowel disease?. Nutrition journal. 2019 Dec;18(1):1-8. 8. de Silva PS, Luben R, Shrestha SS, Khaw KT, Hart AR. Dietary arachidonic and oleic acid intake in ulcerative colitis etiology: a prospective cohort study using 7-day food diaries. European journal of gastroenterology & hepatology. 2014 Jan 1;26(1):11-8 9. Peters V, Bolte L, Schuttert E, Andreu-Sánchez S, Dijkstra G, Weersma R, Campmans-Kuijpers M. Western and carnivorous dietary patterns are associated with greater likelihood of IBD development in a large prospective population-based cohort. Journal of Crohn's and Colitis. 2022 Jun;16(6):931-9 10. Khalili H, Hakansson N, Casey K, Lopes E, Ludvigsson JF, Chan AT, Chan SS, Olen O, Wolk A. Diet Quality and Risk of Older-Onset Crohn’s Disease and Ulcerative Colitis. Journal of Crohn's and Colitis. 2022 Dec 15. | Not relevant exposure |
| 1. Meyer A, Dong C, Casagrande C, Chan SS, Huybrechts I, Nicolas G, Rauber F, Levy RB, Millett C, Oldenburg B, Weiderpass E. Food processing and risk of Crohn’s disease and ulcerative colitis: A European Prospective Cohort Study. Clinical Gastroenterology and Hepatology. 2022 Oct 12. 2. Hart AR, Luben R, Olsen A, Tjonneland A, Linseisen J, Nagel G, Berglund G, Lindgren S, Grip O, Key T, Appleby P. Diet in the aetiology of ulcerative colitis: a European prospective cohort study. Digestion. 2008;77(1):57-64. | Article with similar exposure and outcome variable (Similar population) |
| 1. Maconi G, Ardizzone S, Cucino C, Bezzio C, Russo AG, Bianchi Porro G. Pre-illness changes in dietary habits and diet as a risk factor for inflammatory bowel disease: a case-control study. World J Gastroenterol. 2010 Sep 14;16(34):4297-304. 2. Rashvand S, Somi MH, Rashidkhani B, Hekmatdoost A. Dietary protein intakes and risk of ulcerative colitis. Medical Journal of the Islamic Republic of Iran. 2015;29:253. 3. Preda C, Manuc T, Chifulescu AE, Istratescu D, Louis E, Baicus C, Sandra I, Diculescu M, Reenaers C, Van Kemseke C, Nitescu M. Diet as an environmental trigger in inflammatory bowel disease: A retrospective comparative study in two European cohorts. Revista Espanola de Enfermedades Digestivas. 2020;112(6). 4. Farsi F, Tahvilian N, Heydarian A, Karimi S, Ebrahimi S, Ebrahimi‐Daryani N, Tabataba‐Vakili S, Heshmati J, Mokhtare M. Evaluating macro‐and micronutrients and food groups intake with the risk of developing inflammatory bowel disease: Is there any association?. Food Science & Nutrition. 2022 Nov;10(11):3920-30. 5. Ng SC, Tang W, Leong RW, Chen M, Ko Y, Studd C, Niewiadomski O, Bell S, Kamm MA, de Silva HJ, Kasturiratne A. Environmental risk factors in inflammatory bowel disease: a population-based case-control study in Asia-Pacific. Gut. 2015 Jul 1;64(7):1063-71. 6. Naomasa S, Suminori K, Kenji W, Yoshihiro F, Masamichi S, Takashi S, Yutaka I, Yoshihiro M, Satoshi S, Kazushi O, Gen K. Dietary Risk Factors for Inflammatory Bowel Disease. Inflammatory Bowel Diseases. 2005;11(2):154-63. 7. Chen B, Han Z, Geng L. Mendelian randomization analysis reveals causal effects of food intakes on inflammatory bowel disease risk. Frontiers in Immunology. 2022;13. 8. Elmaliklis IN, Liveri A, Ntelis B, Paraskeva K, Goulis I, Koutelidakis AE. Increased functional foods’ consumption and Mediterranean diet adherence may have a protective effect in the appearance of gastrointestinal diseases: a case–control study. Medicines. 2019 Apr 9;6(2):50. 9. Kobayashi Y, Ohfuji S, Kondo K, Fukushima W, Sasaki S, Kamata N, Yamagami H, Fujiwara Y, Suzuki Y, Hirota Y. Association of dietary fatty acid intake with the development of ulcerative colitis: a multicenter case-control study in Japan. Inflammatory Bowel Diseases. 2021 May;27(5):617-28. 10. Khademi Z, Saneei P, Hassanzadeh-Keshteli A, Daghaghzadeh H, Tavakkoli H, Adibi P, Esmaillzadeh A. Association between inflammatory potential of the diet and ulcerative colitis: a case-control study. Frontiers in nutrition. 2021 Feb 10;7:602090. 11. Han MK, Anderson R, Viennois E, Merlin D. Examination of food consumption in United States adults and the prevalence of inflammatory bowel disease using National Health Interview Survey 2015. PLoS One. 2020 Apr 23;15(4):e0232157. 12. DeClercq V, Langille MG, Van Limbergen J. Differences in adiposity and diet quality among individuals with inflammatory bowel disease in Eastern Canada. PloS one. 2018 Jul 19;13(7):e0200580. | Case-control or cross-sectional studies |

**Table S4.** Characteristics of prospective cohort studies in a meta-analysis of total protein, animal protein, and animal protein sources with risk of inflammatory bowel diseases.

| **Author**  **(Country, year)** | **Study name** | **Age**  **(year)^1^** | **Follow up** | **Participants/** **Cases** | **Dietary**  **assessment**  **method** | **Assessment of**  **IBD** | **Outcome** | **Exposure type** | | **Protein (comparison)** | | **Covariate^2^** |
| --- | --- | --- | --- | --- | --- | --- | --- | --- | --- | --- | --- | --- |
|  |  |  |  |  |  |  |  |  |  | **Lowest (ref.)** | **Highest** |  |
| Rubin et al  (Denmark, 2020) (1) | DCH | 56 | 5 y | 54567/542 | FFQ | ICD | IBD | | Total meat | T1 | T3 | 1,2,3,4,5,6,7,8 |
| Dong et al,  (Eight European countries, 2022) (2) | EPIC cohort | 51.8 | 16 y | 413590/595 | FFQ | Self-administered questionnaires | CD, UC | | Total protein, Animal protein, Total meat, Red meat, Poultry, Processed meat, Fish, Egg, Dairy | Q1 | Q4 | 2,4,5,6,9,10 |
| Julià et al.  (Spain, 2021) (3) | IMIDC | 41.87 | 5 y | 11308/3353 | FFQ | DAS28 and clinician | CD, UC | | Total meat, Fish, Egg, Dairy | Continues (g/d) | | 1,2,5,6,9,11,12 |
| Ananthakrishnan et al.  (USA, 2015) (4) | NHS-II | 36 | 9 y | 84803/173 | FFQ | Self-reported and medical report | CD, UC | | Total protein, Animal protein, Fish | Q1 | Q4 | 1,5,9,10,13,14,15,16,17 |
| Narula et al.  (21countries, 2021) (5) | PURE | 52.5 | 9.7 y | 116087/476 | FFQ | Self-reported questionnaires | IBD, CD, UC | | Red meat, Poultry, Processed meat, Dairy | T1 | T3 | 2,3,4,5,6,10,11 |
| Cohen et al.  (USA, 2013) (6) | CCFA | 43.9 | 1 y | 4001/1221 | FFQ | Self-reported questionnaires | CD, UC | | Red meat, Processed meat, Dairy | Continues (g/d) | | 1,2,18 |
| Khalili et al.  (Sweden, 2020) (7) | SMC | 61 | 17 y | 83147/559 | FFQ | ICD | CD, UC | | Red meat, Fish, Dairy | Q1 | Q4 | 1,2,3,5,10 |
| Khalili et al.  (USA, 2017) (8) | NHS-I & NHS-II | 43 | 27 y | 3038049/582 | FFQ | Self-reported confirm medical records | CD, UC | | Red meat, Processed meat | Q1 | Q5 | 1,5,9,10,13,14,15,16,17 |
| Larsson et al.  (Sweden, 2016) (9) | MDC | 58 | 22 y | 28095/135 | Dietary habits | Endoscopy | IBD | | Total protein | Q1 | Q4 | 1,2,5 |
| Opstelten et al.  (European (12 centers), 2016) (10) | EPIC | 55.4 | 19 y | 401326/354 | FFQ | Medical notes | CD, UC | | Dairy | Q1 | Q4 | 3,5 |
| Jantchou et al.  (France, 2010) (11) | E3N | 56.3 | 10.4 y | 67581/77 | Dietary habits | Self-reported questionnaires | IBD | | Total protein, Animal protein, Total meat, Fish, Egg, Dairy | T1 | T3 | 3 |

Abbreviation: Ref, reference; RR, risk ratio; CI, confidence interval; y, year; T, tertiles; Q, quartiles or quintiles; FFQ, food frequency questionnaires; IBD, inflammatory bowel diseases; CD, Crohn’s disease; UC, ulcerative colitis; DCH, Danish Diet, Cancer and Health cohort; ICD, International Classification of Diseases; EPIC, European Prospective Investigation into Cancer and Nutrition; IMIDC, Immune-Mediated Inflammatory Diseases Consortium; DAS28, Disease Activity Score for 28 joints; NHSII, Nurses’ Health Study II; PURE, Prospective Urban Rural Epidemiology; CCFA, Crohn’s and Colitis Foundation of America; SMC, Swedish Men and Swedish Mammography Cohort; MDC, The Malmö Diet and Cancer; E3N, Etude É pid é miologique des femmes de la Mutuelle G é n é rale de l ’ Education Nationale.

^1^ values are mean (range)

^2^Adjustments: age (1), sex (2), energy (3), alcohol intake (4), smoking status (5), education (6), civil status (7), co-morbidity (8), physical activity (9), body mass index (10), geographical region (11), season of the year (12), oral contraceptive use (13), menopausal status and post-menopausal hormone use (14), non-steroidal anti-inflammatory drug use (15), dietary fiber (16), vitamin D intake (17), prior surgery (18).

**Table S5.** Subgroup analyses of dietary red meat intake and risk of inflammatory bowel diseases (Highest vs. lowest category meta-analysis).

| **Sub-grouped by** | **Number of effect sizes** | **Relative Risk (95%CI)** | **P value^1^** | **I^2^ (%)** | **P for heterogeneity** | **P for between**  **subgroup heterogeneity^2^** |
| --- | --- | --- | --- | --- | --- | --- |
| All studies | 6 | 1.10 (0.97, 1.25) | 0.136 | 55.6 | 0.013 |  |
| Region | | | | | | **0.005** |
| US | 4 | 0.92 (0.80, 1.05) | 0.196 | 0.0 | 0.424 |  |
| Europe | 7 | 1.25 (1.14, 1.37) | <0.001 | 0.0 | 0.433 |  |
| Follow-up duration | | | | | | **0.782** |
| < 10 years | 5 | 1.12 (0.94, 1.32) | 0.208 | 70.2 | 0.009 |  |
| ≥ 10 years | 6 | 1.07 (0.88, 1.31) | 0.492 | 42.2 | 0.124 |  |
| Number of participants | | | | | | **0.985** |
| < 100000 | 6 | 1.10 (0.95, 1.28) | 0.198 | 64.1 | 0.016 |  |
| ≥ 100000 | 4 | 1.09 (0.84, 1.43) | 0.520 | 53.1 | 0.074 |  |
| Number of cases | | | | | | **0.824** |
| < 500 | 7 | 1.08 (0.92, 1.28) | 0.344 | 31.0 | 0.192 |  |
| ≥ 500 | 4 | 1.11 (0.91, 1.36) | 0.298 | 77.6 | 0.004 |  |
| Dietary assessment | | | | | | **-** |
| FFQ | 11 | 1.10 (0.97, 1.24) | 0.136 | 55.6 | 0.022 |  |
| Diet history questionnaire | 0 | - | - | - | - |  |
| Case ascertainment | | | | | | **0.898** |
| Self-reported | 5 | 1.06 (0.88, 1.28) | 0.549 | 48.1 | 0.103 |  |
| Medical records | 4 | 1.15 (0.93, 1.43) | 0.205 | 65.5 | 0.034 |  |
| ICD | 2 | 1.06 (0.87, 1.29) | 0.543 | 0.0 | 0.798 |  |
| Gender |  |  |  |  |  | **0.207** |
| Both | 9 | 1.13 (1.00, 1.28) | 0.047 | 53.3 | 0.029 |  |
| Women | 2 | 0.87 (0.54, 1.39) | 0.559 | 59.4 | 0.117 |  |
| Adjustment for confounders | | | | | | |
| Energy intake | | | | | | **0.316** |
| Yes | 5 | 1.03 (0.89, 1.19) | 0.731 | 0.7 | 0.402 |  |
| No | 6 | 1.16 (0.97, 1.39) | 0.099 | 69.3 | 0.006 |  |
| Physical activity | | | | | | **0.028** |
| Yes | 6 | 1.21 (1.01, 1.44) | 0.038 | 52.9 | 0.060 |  |
| No | 5 | 0.99 (0.89, 1.11) | 0.872 | 0.0 | 0.710 |  |
| Alcohol intake | | | | | | **0.321** |
| Yes | 3 | 1.26 (0.98, 1.62) | 0.069 | 17.7 | 0.297 |  |
| No | 8 | 1.06 (0.92, 1.23) | 0.008 | 63.2 | 0.008 |  |
| Smoking status | | | | | | **0.066** |
| Yes | 9 | 1.17 (1.03, 1.33) | 0.014 | 39.7 | 0.103 |  |
| No | 2 | 0.93 (0.80, 1.08) | 0.313 | 0.0 | 0.636 |  |
| BMI | | | | | | **0.824** |
| Yes | 7 | 1.08 (0.92, 1.28) | 0.344 | 31.0 | 0.192 |  |
| No | 4 | 1.11 (0.91, 1.36) | 0.298 | 77.6 | 0.004 |  |
| Sex | | | | | | **0.207** |
| Yes | 9 | 1.13 (1.00, 1.28) | 0.047 | 53.3 | 0.029 |  |
| No | 2 | 0.87 (0.54, 1.39) | 0.559 | 59.4 | 0.117 |  |

FFQ, Food Frequency Questionnaire; ICD, International Classification of Diseases; BMI, body mass index.

^1^ P heterogeneity within subgroup.

^2^ P heterogeneity between subgroups using meta-regression analysis.

**Table S6.** Subgroup analyses of dietary dairy intake and risk of inflammatory bowel diseases (Highest vs. lowest category meta-analysis).

| **Sub-grouped by** | **Number of effect sizes** | **Relative Risk (95%CI)** | **P value^1^** | **I^2^ (%)** | **P for heterogeneity** | **P for between**  **subgroup heterogeneity^2^** |
| --- | --- | --- | --- | --- | --- | --- |
| All studies |  |  |  |  |  |  |
| Region | | | | | | **0.925** |
| US | 2 | 0.81 (0.68, 0.98) | 0.028 | 0.0 | 0.510 |  |
| Europe | 10 | 0.80 (0.70, 0.90) | 0.002 | 46.4 | 0.052 |  |
| Follow-up duration | | | | | | **0.532** |
| < 10 years | 5 | 0.84 (0.73, 0.96) | 0.013 | 40.0 | 0.155 |  |
| ≥ 10 years | 7 | 0.76 (0.62, 0.92) | 0.006 | 41.8 | 0.112 |  |
| Number of participants | | | | | | **0.254** |
| < 100000 | 7 | 0.77 (0.68, 0.88) | <0.001 | 42.0 | 0.111 |  |
| ≥ 100000 | 5 | 0.91 (0.74, 1.11) | 0.345 | 14.9 | 0.320 |  |
| Number of cases | | | | | | **0.704** |
| < 500 | 8 | 0.80 (0.65, 0.99) | 0.045 | 55.4 | 0.028 |  |
| ≥ 500 | 4 | 0.79 (0.71, 0.88) | <0.001 | 0.0 | 0.734 |  |
| Dietary assessment | | | | | | **0.692** |
| FFQ | 11 | 0.80 (0.71, 0.90) | <0.001 | 41.1 | 0.075 |  |
| Diet history questionnaire | 1 | 0.94 (0.53, 1.67) | 0.833 | - | - |  |
| Case ascertainment | | | | | | **0.120** |
| Self-reported | 6 | 0.88 (0.77, 1.01) | 0.070 | 0.0 | 0.458 |  |
| Medical records | 4 | 0.77 (0.68, 0.88) | <0.001 | 0.0 | 0.743 |  |
| ICD | 2 | 0.57 (0.26, 1.24) | 0.157 | 87.8 | 0.004 |  |
| Gender |  |  |  |  |  | **0.692** |
| Both | 11 | 0.80 (0.71, 0.90) | <0.001 | 41.1 | 0.075 |  |
| Women | 1 | 0.94 (0.53, 1.67) | 0.833 | - | - |  |
| Adjustment for confounders | | | | | | |
| Energy intake | | | | | | **0.954** |
| Yes | 6 | 0.77 (0.57, 1.05) | 0.095 | 67.7 | 0.009 |  |
| No | 6 | 0.80 (0.73, 0.89) | <0.001 | 0.0 | 0.880 |  |
| Physical activity | | | | | | **0.910** |
| Yes | 4 | 0.80 (0.71, 0.90) | <0.001 | 0.0 | 0.728 |  |
| No | 6 | 0.80 (0.66, 0.97) | 0.023 | 56.0 | 0.026 |  |
| Alcohol intake | | | | | | **0.126** |
| Yes | 3 | 0.98 (0.77, 1.24) | 0.845 | 23.0 | 0.273 |  |
| No | 9 | 0.77 (0.69, 0.86) | <0.001 | 26.5 | 0.208 |  |
| Smoking status | | | | | | **0.817** |
| Yes | 9 | 0.80 (0.69, 0.92) | 0.003 | 51.5 | 0.036 |  |
| No | 3 | 0.83 (0.69, 0.98) | 0.031 | 0.0 | 0.722 |  |
| BMI | | | | | | **0.656** |
| Yes | 5 | 0.81 (0.60, 1.08) | 0.147 | 72.7 | 0.005 |  |
| No | 7 | 0.79 (0.71, 0.88) | <0.001 | 0.0 | 0.897 |  |
| Sex | | | | | | **0.919** |
| Yes | 9 | 0.81 (0.71, 0.92) | 0.002 | 50.9 | 0.039 |  |
| No | 3 | 0.79 (0.57, 1.09) | 0.146 | 0.0 | 0.622 |  |

FFQ, Food Frequency Questionnaire; ICD, International Classification of Diseases; BMI, body mass index.

^1^ P heterogeneity within subgroup.

^2^ P heterogeneity between subgroups using meta-regression analysis.

| **Table S7: GRADE evidence table for the association of total protein, animal protein and animal protein sources with risk of inflammatory bowel diseases.** | | | | | | | | | | | | |
| --- | --- | --- | --- | --- | --- | --- | --- | --- | --- | --- | --- | --- |
| Certainty assessment | | | | | | | No of patients | | Effect | | Certainty | Importance |
| No of studies | Design | Risk of bias | Inconsistency | Indirectness | Imprecision | Other  considerations | Participants | Cases (%) | Relative  (95% CI) | Absolute  (95% CI) |  |  |
| Total protein | | | | | | | | | | | | |
| 4 | observational studies | serious^a^ | not serious | not serious | serious^b^ | none | 594069 | 980 (0.2%) | **RR 1.22** (0.88 to 1.69) | **0 fewer per 1,000** (from 0 fewer to 1 more) | ⨁⨁◯◯ Low | IMPORTANT |
| Animal protein | | | | | | | | | | | | |
| 3 | observational studies | serious^a^ | serious^c^ | not serious | serious^b^ | none | 565974 | 845 (0.1%) | **RR 1.23** (0.81 to 1.86) | **0 fewer per 1,000** (from 0 fewer to 1 more) | ⨁◯◯◯ Very low | IMPORTANT |
| Red meat | | | | | | | | | | | | |
| 6 | observational studies | serious^a^ | not serious | not serious | serious^d^ | none | 3666182 | 7383 (0.2%) | **RR 1.10** (0.97 to 1.25) | **0 fewer per 1,000** (from 0 fewer to 1 more) | ⨁⨁◯◯ Low | IMPORTANT |
| Processed meat | | | | | | | | | | | | |
| 5 | observational studies | serious^a^ | serious^e^ | not serious | serious^d^ | none | 3583035 | 6824 (0.2%) | **RR 1.09** (0.94 to 1.26) | **0 fewer per 1,000** (from 0 fewer to 0 fewer) | ⨁◯◯◯ Very low | IMPORTANT |
| Poultry | | | | | | | | | | | | |
| 2 | observational studies | serious^a^ | not serious | serious^f^ | serious^d^ | none | 529677 | 1071 (0.2%) | **RR 1.18** (0.88 to 1.59) | **0 fewer per 1,000** (from 0 fewer to 1 more) | ⨁◯◯◯ Very low | IMPORTANT |
| Fish | | | | | | | | | | | | |
| 5 | observational studies | serious^a^ | not serious | not serious | serious^d^ | none | 660429 | 4757 (0.7%) | **RR 1.03** (0.92 to 1.15) | **0 fewer per 1,000** (from 1 fewer to 1 more) | ⨁⨁◯◯ Low | IMPORTANT |
| Total meat | | | | | | | | | | | | |
| 4 | observational studies | serious^a^ | serious^g^ | not serious | serious^a^ | dose response gradient | 535738 | 1214 (0.2%) | **RR 1.24** (0.90 to 1.70) | **1 more per 1,000** (from 0 fewer to 2 more) | ⨁⨁◯◯ Low | IMPORTANT |
| Dairy | | | | | | | | | | | | |
| 7 | observational studies | serious^a^ | not serious | not serious | not serious | none | 1097040 | 7232 (0.7%) | **RR 0.81** (0.72 to 0.90) | **1 fewer per 1,000** (from 2 fewer to 1 fewer) | ⨁⨁⨁◯ Moderate | IMPORTANT |
| Egg | | | | | | | | | | | | |
| 3 | observational studies | serious^a^ | not serious | not serious | serious^d^ | none | 492497 | 4025 (0.8%) | **RR 0.92** (0.81 to 1.04) | **1 fewer per 1,000** (from 2 fewer to 0 fewer) | ⨁⨁◯◯ Low | IMPORTANT |

CI: confidence interval; RR: risk ratio

Explanations

a. Downgraded since most studies judged as serious risk of bias based on ROBINS-I were included in the meta-analysis and residual confounding cannot be ruled out

b. Serious imprecision since the 95%CI include the null value and upper bound of the 95%CI was>1.10. Downgraded.

c. Serious inconsistency since I^2^ = 62.5%. Downgraded.

d. Serious imprecision since the 95% include the null value and bound were <0.90 and >1.10. Downgraded.

e. Serious inconsistency since I^2^ = 61.4%. Downgraded.

f. Serious indirectness since only two cohorts were available. Downgraded.

g. Serious inconsistency since I^2^ = 66.8%. Downgraded.

**Reference:**

1. Rubin KH, Rasmussen NF, Petersen I, Kopp TI, Stenager E, Magyari M, Hetland ML, Bygum A, Glintborg B, Andersen VJIJoMS. Intake of dietary fibre, red and processed meat and risk of late-onset Chronic Inflammatory Diseases: A prospective Danish study on the “diet, cancer and health” cohort. 2020;17(16):2487.

2. Dong C, Chan SS, Jantchou P, Racine A, Oldenburg B, Weiderpass E, Heath AK, Tong TY, Tjønneland A, Kyrø CJJoCs, et al. Meat intake is associated with a higher risk of ulcerative colitis in a large European prospective cohort studyø. 2022;16(8):1187-96.

3. Julià A, Martínez-Mateu SH, Domènech E, Cañete JD, Ferrándiz C, Tornero J, Gisbert JP, Fernández-Nebro A, Daudén E, Barreiro-de Acosta MJEjocn. Food groups associated with immune-mediated inflammatory diseases: a Mendelian randomization and disease severity study. 2021;75(9):1368-82.

4. Ananthakrishnan AN, Khalili H, Song M, Higuchi LM, Richter JM, Nimptsch K, Wu K, Chan ATJIBD. High school diet and risk of Crohn's disease and ulcerative colitis. 2015;21(10):2311-9.

5. Narula N, Wong EC, Dehghan M, Mente A, Rangarajan S, Lanas F, Lopez-Jaramillo P, Rohatgi P, Lakshmi P, Varma RPJB. Association of ultra-processed food intake with risk of inflammatory bowel disease: prospective cohort study. 2021;374.

6. Cohen AB, Lee D, Long MD, Kappelman MD, Martin CF, Sandler RS, Lewis JDJDd, sciences. Dietary patterns and self-reported associations of diet with symptoms of inflammatory bowel disease. 2013;58:1322-8.

7. Khalili H, Håkansson N, Chan SS, Chen Y, Lochhead P, Ludvigsson JF, Chan AT, Hart AR, Olén O, Wolk AJG. Adherence to a Mediterranean diet is associated with a lower risk of later-onset Crohn’s disease: Results from two large prospective cohort studies. 2020;69(9):1637-44.

8. Khalili H, de Silva PS, Ananthakrishnan AN, Lochhead P, Joshi A, Garber JJ, Richter JR, Sauk J, Chan ATJIbd. Dietary iron and heme iron consumption, genetic susceptibility, and risk of Crohn's disease and ulcerative colitis. 2017;23(7):1088-95.

9. Larsson J, Sonestedt E, Ohlsson B, Manjer J, Sjöberg KJEjocn. The association between the intake of specific dietary components and lifestyle factors and microscopic colitis. 2016;70(11):1309-17.

10. Opstelten JL, Leenders M, Dik VK, Chan SS, Van Schaik FD, Khaw K-T, Luben R, Hallmans G, Karling P, Lindgren SJIbd. Dairy products, dietary calcium, and risk of inflammatory bowel disease: results from a European prospective cohort investigation. 2016;22(6):1403-11.

11. Jantchou P, Morois S, Clavel-Chapelon F, Boutron-Ruault M-C, Carbonnel FJOjotACoG, ACG. Animal protein intake and risk of inflammatory bowel disease: The E3N prospective study. 2010;105(10):2195-201.
